# The Clinicopathological Characteristics and Prognoses of dMMR Gastric Adenocarcinoma Patients

**DOI:** 10.1155/2021/4269781

**Published:** 2021-12-09

**Authors:** Jie Wang, Yanfeng Xi, Jian Zhao, Xuetong Rong, Weidong Lu, Yusheng Wang

**Affiliations:** ^1^Shanxi Medical University, Taiyuan, Shanxi 030001, China; ^2^Department of Pathology, Affiliated Cancer Hospital of Shanxi Medical University, Taiyuan, Shanxi 030013, China; ^3^Department of Digestive, Affiliated Cancer Hospital of Shanxi Medical University, Taiyuan, Shanxi 030013, China

## Abstract

**Background:**

Few studies on the clinicopathological features and prognosis of DNA mismatch repair deficiency (dMMR) gastric cancer (GC) have been reported, and no clear conclusions have been drawn about the factors affecting the prognosis of dMMR GC. The aim of this study was to explore the clinicopathological characteristics and prognoses of dMMR GC patients.

**Methods:**

From May 2011 to November 2020, GC patients who underwent surgery with dMMR confirmed by immunohistochemistry (IHC) at the Affiliated Cancer Hospital of Shanxi Medical University were selected. The patients' clinical and pathological data were collected. The recurrence-free survival (RFS) and overall survival (OS) rates of the patients were determined through follow-up. SPSS 26.0 was used to analyze the patients' clinicopathological features and prognoses.

**Results:**

A total of 162 dMMR GC patients met the inclusion criteria, and the median age was 63.5 years (32–89 years). dMMR GC was more common in males (65% vs. 35%), and most of the cases were stage II (the prevalence of stage I was 22%, that of stage II was 43%, that of stage III was 30%, and that of stage IV was 5%). Most of the lesions were located in the antrum (49%), followed by the cardia (25%). PMS2 and MLH1 (57%) deficiency was most common. Kaplan–Meier analysis showed that factors related to OS were family history (*P* = 0.048), number of lymph node (LN) metastases (*P* < 0.001), vascular tumor thrombus (*P* < 0.001), HER2 expression status (*P* = 0.025), and clinical stage (*P* < 0.001). The factors related to RFS included vascular tumor thrombus (*P* < 0.001), number of LN metastases (*P* < 0.001), and clinical stage (*P* < 0.001).

**Conclusion:**

In this study, dMMR GC was more common in men, and the median age was 63.5 years. Most of the lesions were in the antrum and showed the combined deletion of MLH1 and PMS2. dMMR GC patients tended to be early stage, and the prognosis of those with early-stage GC was better. dMMR GC patients with vascular tumor thrombus or >6 LN metastases had a high recurrence rate and poor survival outcome.

## 1. Introduction

According to data released by the Cancer Statistics Center in 2020, there are 1.089 million new cases of gastric cancer (GC) and 769,000 GC-related deaths worldwide each year; thus, GC ranks fifth and fourth in incidence rate and mortality rate, respectively, among malignancies [1]. China accounts for 44% of yearly GC cases worldwide, and most patients are diagnosed in the advanced stages with poor prognoses. After combined-modality therapy, the median survival period is only 11.4 months, and the 5-year survival rate is 20%–50% [2]. In a 2014 TCGA study, GC was divided into the following 4 molecular subtypes: Epstein–Barr virus-positive, microsatellite instability (MSI), chromosomal unstable, and genomic stable [3]. The MSI subtype is a pathological condition caused by the deletion of mismatch repair (MMR) genes (MLH1, MSH2, MSH6, and PMS2) or an EPCAM gene mutation (which results in MSH2 gene silencing). These aberrations lead to mismatch repair protein deficiency (DNA mismatch repair deficiency (dMMR)). dMMR leads to mistakes in DNA replication that are difficult to repair, eventually causing abnormal cell proliferation, differentiation, and tumorigenesis. The microsatellite status can be evaluated by PCR or next-generation sequencing (NGS). Depending on the results, tumors are classified as microsatellite stable (MSS), MSI-low, or MSI-high (MSI-H). At present, immunohistochemical staining (IHC) of four mismatch repair proteins (MLH1, MSH2, MSH6, and PMS2) is the primary clinical screening method for dMMR/MSI-H status. The consistency rate between IHC and MSI results is high, reaching 90%–95% [4].

Microsatellite status plays an important role in the diagnosis and treatment of malignant tumors [5], especially colorectal cancer (CRC). Microsatellite status can predict the survival prognosis, determine whether patients with stage II colorectal cancer can benefit from 5-Fu treatment after surgery, and determine whether patients with advanced CRC can benefit from single-agent immunotherapy [6, 7]. There have been many studies on the clinicopathological characteristics of dMMR CRC in recent years, and significant results have been obtained [8, 9]. Data analyses are still lacking regarding the clinicopathological characteristics and prognoses of dMMR GC patients [10, 11]. Some dMMR GC patients have germline mutations in MMR genes, also known as Lynch syndrome (LS) type II, with obvious family histories and genetic effects [12]. Whether the survival outcomes of these patients are similar to those of patients with sporadic disease is also worthy of discussion [13]. Therefore, we retrospectively collected the clinical and pathological data of dMMR GC patients diagnosed at the Affiliated Cancer Hospital of Shanxi Medical University over the past 10 years and analyzed the correlation between dMMR status and prognosis. The results are reported in this article.

## 2. Methods

### 2.1. Clinical and Pathological Data

Retrospective study data were obtained from the Affiliated Cancer Hospital of Shanxi Medical University. Pathological data of patients who underwent gastrectomy for GC from May 2011 to November 2020 and were confirmed as dMMR by IHC staining were obtained from the pathology department, regardless of TNM stage or whether systemic treatment was administered. The clinical data of patients were further collected from the Medical Record Room, including sex, age, family history, tumor location, metastasis status, clinical stage, single or multiple lesion presence, vascular tumor thrombus presence, nerve infiltration, and HER2 expression status. The recurrence and survival rates of the enrolled patients were determined through follow-up. This study was approved by the Ethics Committee of the Affiliated Cancer Hospital of Shanxi Medical University.

#### 2.1.1. Inclusion Criteria

The study inclusion criteria were as follows: (1) patients who underwent any surgical treatment (including radical or palliative resection); (2) patients of all TNM stages, regardless of the presence of metastases or not; (3) patients with gastric or gastroesophageal junction adenocarcinoma; and (4) patients who did or did not undergo systemic treatment.

#### 2.1.2. Exclusion Criteria

The exclusion criteria were as follows: (1) patients without surgical treatment or who underwent surgical treatment at other hospitals; (2) patients with other forms of gastric or gastroesophageal junction cancer except adenocarcinoma; and (3) patients with incomplete pathological information. In addition, patients with pMMR (DNA mismatch repair proficiency) confirmed by IHC were excluded.

### 2.2. MMR IHC and Interpretation Standards

The interpretation of MMR protein loss was performed using Leica's automatic IHC staining machine to detect the expression of the MMR proteins MLH1, PMS2, MSH2, and MSH6 in resected GC specimens. The staining was performed according to the set machine program. The criteria for determining successful staining were the staining of tumor cell nuclei and the positive staining of control cell nuclei in each section. The internal control included normal intestinal mucosa, tumor stromal cells, and inflammatory cells. If the internal control was positive and the cancer cells were completely negative (i.e., there was no nuclear staining), protein expression was considered absent. If the internal control and tumor cells were completely negative, the finding was considered a false negative. Two experienced pathologists at our hospital evaluated all stained sections. If their results were different, the samples were reanalyzed.

### 2.3. HER2 IHC and Interpretation Standards

HER2 IHC staining was performed on surgical resection specimens. First, the specimens were pretreated, including fixation, tissue dehydration and embedding, wax block selection, slicing, and dewaxing. Then, ROCHE's fully automated dyeing machine was used to dye the specimens according to the set procedure. Positive, negative, and blank controls were included for each test. The HER2 IHC staining results in GC were interpreted by experienced pathologists. 0 indicated no response or <10% tumor cell membrane staining; 1+ indicated ≥10% weak or faint visible membrane staining or only partial cell membrane staining; 2+ indicated ≥10% of tumor cells with weak to moderate complete staining or cell basal and bilateral side membrane staining; 3+ indicated strong staining or ≥10% of tumor cells completely stained or stained at the cell base and both sides of the side membrane; 0 and 1+ were considered negative; 2+ was considered unable to confirm HER2 overexpression; and 3+ indicated HER2 overexpression.

### 2.4. Follow-Up

The follow-up data of the patients included in this study were collected from the follow-up center at the Affiliated Cancer Hospital of Shanxi Medical University. The follow-up data included survival status, time of death or loss to follow-up, time of recurrence and metastasis, and cause of death. The follow-up period ended on April 26, 2021.

### 2.5. Statistical Methods

Overall survival (OS) was defined as the time from enrollment in the study to the date of death or loss to follow-up from any cause. Recurrence-free survival (RFS) was defined as the time from the date of surgery to the date of the diagnosis of disease recurrence, death, or loss to follow-up for any reason. All data were analyzed using SPSS software version 26.0. Quantitative data are described as the mean ± standard deviation or the median and quartile; qualitative data are described as quantities or percentages. The Kaplan–Meier method was used to evaluate OS and RFS outcomes. The log-rank test was used to analyze and compare each subgroup, and results with a *P* value < 0.05 were considered statistically significant. GraphPad Prism 7.0 was used to generate the survival curves.

## 3. Results

### 3.1. Clinicopathological Characteristics

#### 3.1.1. Clinical Features

A total of 162 patients met the inclusion criteria; 105 were male and 57 were female. The average age at the first visit was 62.1 years, and the median age was 63.5 years (32–89 years). Patients with a family history of tumors accounted for 9.3% (15/162) of the study population. Most of the tumors were located in the antrum (82/162, 48%), followed by the cardia. The longest tumor diameter was over 5 cm, and 9 (5.6%) patients had multiple tumors. Single tumors were present in 94.4% (153/162) of the patients. Regarding clinical TNM staging, 22% of the patients had stage I GC, 43% had stage II GC, 30% had stage III, and 5% had stage IV. The number of lymph node (LN) metastases in 64% of the patients was ≤6; 88% underwent tumor resection by laparotomy and 12% by laparoscopy. See Table 1.

#### 3.1.2. Pathological Features

There were 140 patients with Lauren's classification data available, 55 with mixed type, 51 with the intestinal type, and 34 with the diffuse type. Regarding the degree of differentiation, the proportion of low and medium-low differentiation was 34% (55/162), and 30% had medium differentiation (48/162). Among the 162 patients, 62% were negative for vascular tumor thrombus and 38% were positive; 72% were positive for nerve invasion and 28% were negative. Ninety-six percent of the patients had HER2 expression IHC scores below 3 (156/162), and only 2 patients had HER2 scores of 3. See Table 1.

#### 3.1.3. dMMR Status Based on IHC

**Figure 1:**
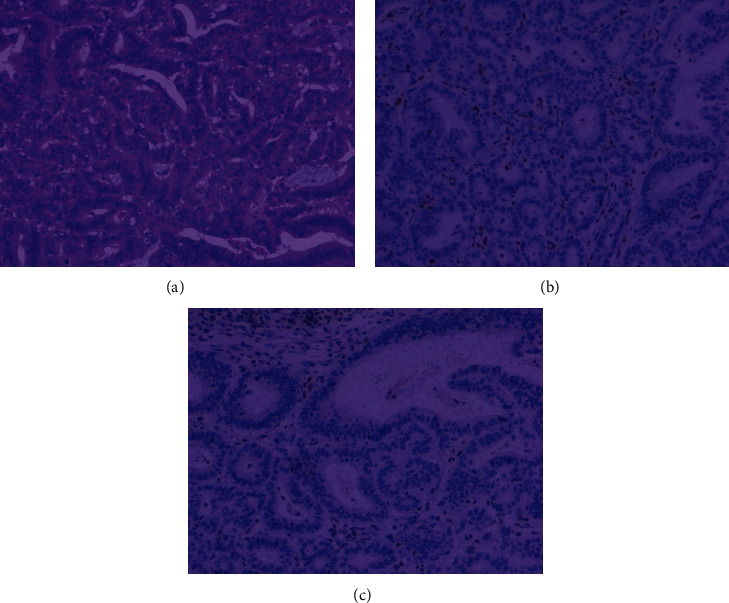
IHC staining for mismatch repair (MMR) proteins. (a) HE staining of GC surgical specimens; (b) the loss of MLH1 protein expression; (c) the loss of PMS2 protein expression.

Of the 162 patients, 57% lacked the expression of MLH1 and PMS2 (93/162), and 27% (44/162) lacked PMS2. MSH6 expression was absent in 6% (9/162) of the patients, 6 lacked MLH1 expression (4%), and MSH2 and MSH6 expression was absent in 3% (5/162) of the patients. Only 2% of the patients lacked MSH2 expression (4/162) deletion (Table 1, Figure 1).

#### 3.1.4. Analysis of Patients with Multiple Primary Tumors

Among the 9 patients with multiple tumors, 3 had a family history of tumors. There were 3 patients with a previous personal history of tumors, all of whom had extra gastric tumors (2 had rectal tumors and 1 had a breast tumor); 7 patients had multiple simultaneous primary tumors, of which 4 tumor lesions were located in the stomach and 3 were extragastric (1 in the right colon, 1 in the small intestine, and 1 in the esophagus). One patient had multiple metachronous primary tumors, and 1 patient had simultaneous and metachronous tumors. Four extragastric tumors were reanalyzed by IHC staining, and the expression status of MMR proteins was consistent with those in the patients' gastric lesions. See Table 2.

### 3.2. Correlation Analysis of Clinicopathological Characteristics, Survival Outcomes, and Recurrence Rates

The follow-up date was up to April 26, 2021. Of the 162 patients enrolled in this study, 31 experienced recurrence and 25 died. The average follow-up time was 20.4 months (0–110 months). The 1-year OS rate was 88%, the 2-year OS rate was 84%, and the 3-year and 5-year OS rates were both 78%. The average time from surgery to recurrence was 19.5 months (0–110 months) among patients who experienced recurrence.

#### 3.2.1. The Variables Related to OS

**Figure 2:**
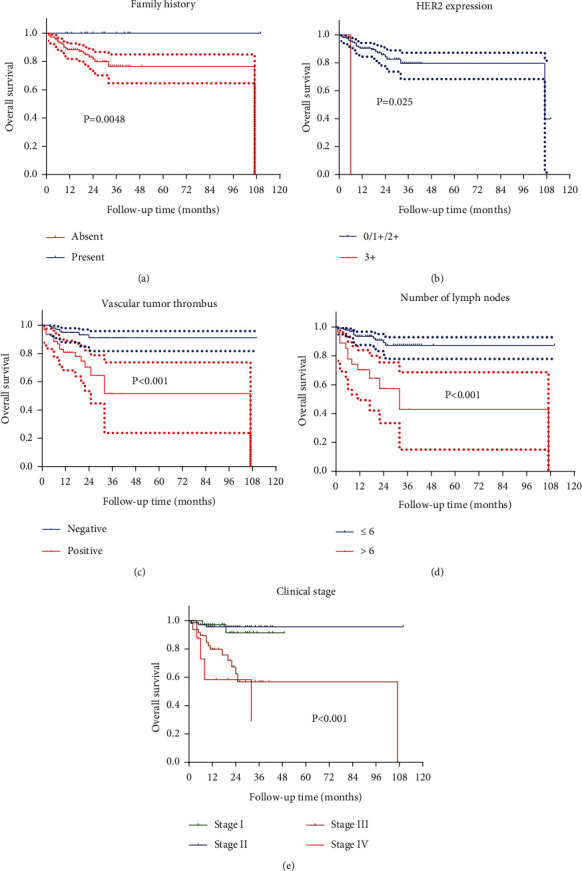
Overall survival of dMMR GC patients depending on (a) family history (present vs. absent, *P* = 0.048), (b) number of LN with metastases (≤6 vs. >6, *P* < 0.001), (c) HER2 expression status (0/1+/2+ vs. 3+, *P* = 0.025), (d) vascular tumor thrombus (negative vs. positive, *P* < 0.001), and (e) clinical stage (*P* < 0.001).

The Kaplan–Meier single-factor survival analysis showed that the OS-related variables were family history (*P* = 0.048), number of LN metastases (*P* < 0.001), presence of vascular tumor thrombus (*P* < 0.001), HER2 expression status (*P* = 0.025), and clinical stage (*P* < 0.001). There was no significant correlation between OS and sex, age, surgical approach, single/multiple lesions, dMMR status, tumor size, differentiation degree, or Lauren's classification. See Figure 2.

#### 3.2.2. The Variables Related to RFS

**Figure 3:**
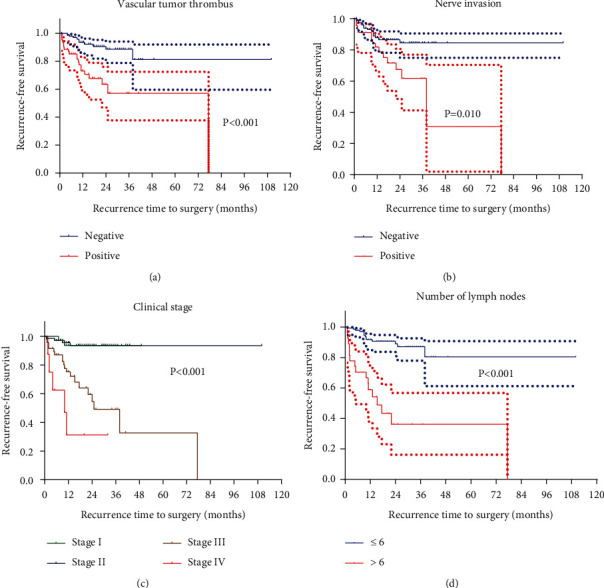
Recurrence-free survival of GC patients depending on (a) vascular tumor thrombus (negative vs. positive, *P* < 0.001), (b) nerve invasion (negative vs. positive, *P* = 0.010), (c) clinical stage (*P* < 0.001), and (d) number of LN with metastases (≤6 vs. >6, *P* < 0.001).

The Kaplan–Meier single-factor survival analysis showed that the variables related to RFS were nerve invasion (*P* = 0.010), vascular tumor thrombus (*P* < 0.001), the number of LN with metastases (*P* < 0.001), and clinical stage (*P* < 0.001). See Figure 3.

#### 3.2.3. Analysis of the Clinical and Pathological Characteristics of the Patients Who Died

**Figure 4:**
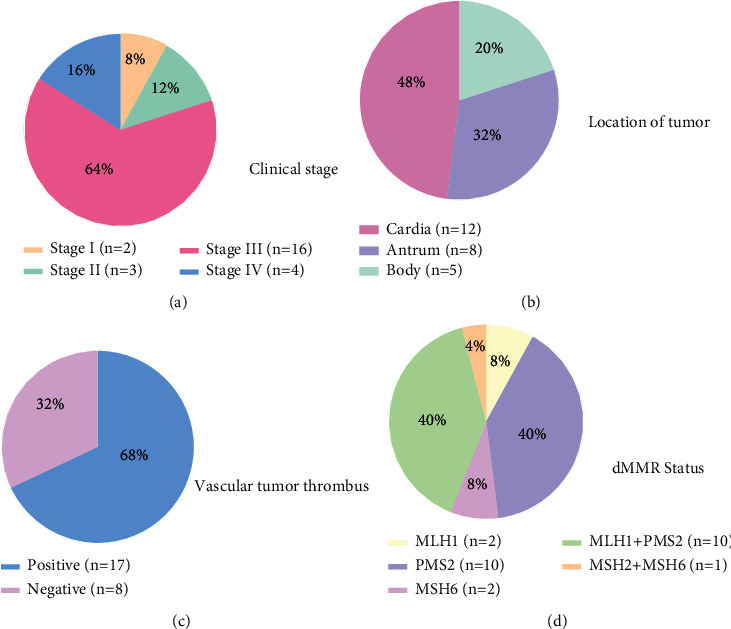
The proportion of (a) patients with clinical stage I-IV GC, (b) tumors located in different gastric areas, (c) patients with vascular tumor thrombus, and (d) dMMR statuses among patients who died (*n* = 25).

Among the 25 patients who died, the median age at first diagnosis was 64 years (range: 48–89 years), and the median survival time was 9 months. Most of the patients were male (64%). There was no family history of tumors. The most common clinical stage was stage III (64%), the most common mismatch repair protein abnormalities were the lack of PMS2 expression only (10/25, 40%) and the lack of MLH1 and PMS2 expression (10/25, 40%), and more of the patients who died had an absence of HER2 expression (Figure 4).

Among the 25 patients who died, 17 (68%) had vascular tumor thrombi, 11 (11/17, 65%) had tumors located in the cardia, 4 (4/17, 24%) had gastric antrum tumors, and 2 had gastric body tumors (2/17, 12%). PMS2 was absent in 7 patients (41%), and MLH1 and PMS2 were absent in 6 patients (35%). There were 13 patients (76%) with clinical stage III disease, 3 patients with stage IV disease, and 1 patient with stage II disease. The patient with stage II disease died 20 days after surgery.

Two deaths occurred within 1 month after surgery. These patients had stage II and stage III disease and were 62 and 72 years of age, respectively, and the former had nerve invasion and vascular tumor thrombus. Ten patients died within six months after surgery. The average age of these patients was 65.2 years. Six of them had gastric antrum tumors, 4 had gastric cardia tumors, 6 had stage III GC, 2 had stage IV GC, 1 had stage I GC (an 89-year-old patient), and 1 had stage II GC (recurrence occurred 2 months after surgery). Vascular tumor thrombi were present in 7 patients.

## 4. Discussion

The incidence of GC is high worldwide and varies regionally. The incidence of GC in East Asia is much higher than that in other regions. Over 700,000 new cases of GC are diagnosed in China each year; thus, China has the highest incidence of GC worldwide [14]. Shanxi Province is a high-risk area for GC in China. The incidence of GC in this region ranks second among all malignant tumors, behind that of lung cancer. The incidence and mortality rate of GC differs based on age, sex, and location. The incidence and mortality rate of GC significantly increases with age. There are also differences in GC risk depending on geographical location. Statistically, the incidence and mortality rates of GC in men are higher than those in women. This study included 162 patients with dMMR GC pathologically diagnosed at Shanxi Cancer Hospital over the past 10 years. The data show that there were more men (65%) affected than women (35%), and the male mortality rate (64%) was also significantly higher than that of women, consistent with China's published data on the incidence and death rate of GC. The median age at first diagnosis in this study was 63.5 years; the most common site for dMMR tumors was the gastric antrum (49%), followed by the cardia (25%); Lauren's classification had no obvious specificity; the most common TNM stage was stage II (43%), followed by stage III (30%). TCGA reports and the results of many other studies [15–17] indicate that the average age of MSI-type GC diagnosis is 72 years, intestinal-type GC is the most common, and TNM staging typically earlier (stage I or II); thus, our findings are different from those of previous studies. This may be due to geographic differences.

Tumors are mainly caused by mutations in driver genes. The normal human body has a functional MMR system that repairs errors in the DNA replication process, thereby maintaining the stability of the genome. The absence of components of the MMR system complicates DNA repair and eventually leads to tumor formation. The human mismatch repair system comprises two protein heterodimers. Under normal circumstances, the MSH heterodimer (Mut-S (MSH2-MSH6) or Mut-Sb (MSH2-MSH3)) first recognizes and binds to the mismatched site during DNA replication. When Mut-S or Mut-Sb recognizes and binds to the mismatched base, it recruits the homodimer Mut-L (MLH1-PMS2) to initiate the repair process. MLH1, PMS2, MSH2, MSH3, and PMS2 are also referred to as MMR proteins. Because MSH3 is rare in humans, solid tumors are most often caused by the loss of one or more of the other 4 MMR proteins. Microsatellites are short genomic tandem repeat sequences, generally 1–6 bp in length, and are present in approximately 10% of human genes. When an insertion-deletion loop (IDL) occurs in human DNA and the length of the microsatellite changes, MSI arises. If a mutation in the MMR gene affects the MMR protein, a repair function defect occurs, and errors in the DNA replication process cannot be repaired, leading to the insertion or deletion of DNA sequences that subsequently cause to MSI. Mutations in the MMR genes can cause MSI, and the occurrence of MSI is not necessarily a result of MMR protein deletion—MSI can also be caused by methylation of the MLH1 promoter. MSI tumors caused by germline mutations in the MMR genes are referred to as Lynch syndrome (LS). LS type II, also known as extraintestinal syndrome, is more common in men with GC. In our study, we found that PMS2 single-point mutations or PMS2-MLH1 dimer mutations are predominant in patients with dMMR GC. Additionally, although Mut-S (MSH2-MSH6) can bind to DNA error replication sites, its repair function cannot be completed. This issue is worthy of further study. In our study, there were 9 patients with multiple primary tumors. The extragastric lesions were mainly in the digestive tract, which is consistent with the pathogenesis of LS. Multiple primary tumor tissues were confirmed by IHC to show a consistent lack of one or more MMR proteins, which supports the homogeneous MMR protein loss in different primary tumor lesions within the same individual. This homogeneity is also a direction worthy of further exploration.

Due to the low rate of early diagnosis, GC diagnosed in the Shanxi Province is typically advanced and unresectable, and the primary treatment is systemic chemotherapy and targeted therapy; thus, GC patients have a poor prognosis. According to data from TCGA, MSI GC accounts for 22% of GC cases, and the prognosis of patients with MSI GC is good. In the era of immunotherapy, the dMMR/MSI-H population is primarily treated with immune checkpoint inhibitors. According to clinical studies, such as the KEYNOTE-061 study of 14 noncolorectal tumor patients with dMMR/MSI-H cancer, the objective response rate (ORR) to pabolizumab as a second-line and subsequent treatment can reach 46%, and that of GC and gastroesophageal junction cancer can reach 56%. The results of the CHECKMATE-649 trial showed that in patients with GC with a CPS score ≥ 5, the ORR to navulizumab combined with chemotherapy reached 68%, which was 48% higher than that of the chemotherapy group [18, 19]. The above studies showed that the efficacy of immunotherapy was higher than that of previous systemic treatment, but some patients with dMMR/MSI-H still had a poor prognosis and it is necessary to determine the factors affecting the prognoses of these patients. The clinicopathological features of dMMR GC patients in the Shanxi Province obtained from our study can be used for the early screening of such patients, potentially leading to improved diagnostics and earlier treatment to improve the curative effect and patient prognosis.

In this study, the 1-year survival rate of dMMR GC patients after surgery reached 88%, and the 5-year survival rate was 78%. According to relevant literature reports, the 5-year survival rate of pMMR GC patients is 20%-50%, which is quite different from the survival rate of the dMMR patients in our study. The higher postoperative survival rate of dMMR patients in our study may be related to the relatively early clinical stage (mostly stage II) at which the patients were first diagnosed, the relatively small number of lymph node metastases present, and the small number of metastases overall at the time of the first diagnosis. The 3-year and 5-year OS rates were both 78%, indicating that the condition of patients with dMMR GC was stable and that the risk for death was significantly reduced at 3 years after surgery. In addition, our study revealed that although dMMR GC is more likely to occur in the gastric antrum, most of the patients who died had tumors located in the cardia. This finding indicates that regardless of the molecular type of GC, tumors of the gastroesophageal junction are associated with worse prognosis GC patient prognosis than tumors located in the gastric antrum and other areas, and the risk for death related to gastroesophageal junction tumors is higher.

The univariate analysis revealed that the overall survival outcome of patients with dMMR GC is related to family history, vascular tumor thrombus, clinical stage, number of lymph node metastases, and strong HER2 expression. In the Cox multivariate regression analysis, only the number of LN metastases was correlated with OS and RFS. This finding suggests that the number of regional lymph node metastases is significantly correlated with disease recurrence and patient survival (*P* < 0.001). Even among patients with early-stage tumors, those with many LN metastases have a substantial risk for recurrence and metastasis after surgery. Previous studies have shown that the recurrence of GC after surgery is significantly related to the number of intraoperative regional LN dissections and the number of positive nodes among these dissected nodes [20–22]. Therefore, to better judge the number of lymph nodes with metastases, it is particularly important to dissect a sufficient number of regional lymph nodes during surgery. In addition, there was a significant correlation between the presence of vascular tumor thrombi and OS and RFS outcome among patients with dMMR GC (*P* < 0.001). In addition, the proportion of patients positive for vascular tumor thrombi positive (68%) among the patients who died was significantly higher than that among the patients who died. The results of many other studies [23, 24] also support that vascular tumor thrombus can be an independent risk factor that affects the risk for postoperative recurrence and death among patients with GC. To reduce the risk for recurrence and death among GC patients and prolong their survival times, the choice of postoperative adjuvant chemotherapy, single-agent immunosuppressive therapy, or adjuvant chemotherapy combined with immunotherapy is worthy of further discussion. Another noteworthy result is that the 15 patients with a family history of tumors in this study all survived long term; even if the patients had stage III GC and experienced relapse after surgery, the survival outcome was better. We did not perform germline genetic testing of these patients and their families, and we cannot exclude LS as a diagnosis in this group of patients. This study evaluated HER2 expression through IHC detection. Previous studies have shown that HER2 IHC detection and FISH gene amplification analyses produce consistent results for 0/1+ and 3+ status; however, the results are ambiguous for 2+ [25]. In this study, patients were divided into 2 groups, and the 2+ group was classified as a weak expression group. Survival analyses have revealed that patients with HER2 overexpression (3+) have a higher risk for death after surgery than those with low expression or no expression of HER2 (0/1+/2+); however, the grouping of 0/1+/2+ and 3+ patients in this study was more stringent. This grouping also reduces the sample size of HER2-overexpression patients as only two patients in this group had HER2 3+ status; thus, the conclusions drawn may have certain limitations and more data are required in support.

The average time to relapse in this study was 19.5 months, and close follow-up (endoscopic monitoring and/or imaging examination) is recommended within 1 to 2 years after surgery. In patients with high-risk factors (such as late clinical stage, >6 lymph node metastases, vascular tumor thrombus, nerve invasion, and HER2 overexpression), the follow-up frequency can be increased to six months or three months. For patients with a family history of tumors, it is recommended to conduct germline genetic testing. If the patients are determined to have LS due to germline gene mutations, it is recommended that their first-degree and second-degree relatives are regularly monitored by digestive endoscopies (EGD), gynecological ultrasounds, and other related examinations. In this study, it was found that dMMR GC in the Chinese population is most commonly caused by MLH1 and PMS2 deletions. NCCN guidelines recommend that MLH1-type LS be monitored by colonoscopy either from 20 to 25 years of age or beginning before the youngest age that colorectal cancer has occurred in the family. Starting from the age of 2–5, the recommended frequency is once every 1–2 years. Colonoscopy monitoring for PMS2-type LS can be postponed until 30–35 years of age. There is no consensus on the frequency of gastroscopy monitoring for people with LS [26, 27]. The German S3 guidelines recommend that EGD monitoring can be initiated for LS patients at the age of 35 years, and the NCCN guidelines recommend that LS patients with high-risk factors (such as digestive system symptoms, a family history of cancer, and Asian ethnicity) can consider EGD monitoring every 3–5 years beginning at the age of 40 years. More research is required to further confirm the preventive effect of gastroscopy monitoring on the occurrence of GC among LS patients and the optimal frequency of gastroscopy monitoring. At present, it is recommended to conduct individualized gastroscopy monitoring according to the patient's wishes, family history, and high-risk factors.

This was a single-cohort, retrospective study and lacked controlled data. MSI status results were lacking due limited time available for this study; thus, it was impossible to determine whether the patients included in the study had germline mutations. In the next step, we will further expand the sample size and perform PCR or NGS on the patients included in the study to clarify their MSI statuses and conduct a study comparing the clinicopathological characteristics of the MSS population with the dMMR population to better understand different MMR statuses and the characteristics and prognoses of patients with GC.

## 5. Conclusion

In this study, dMMR GC was more common in men, and the median age at first diagnosis was 63.5 years. dMMR GC most commonly arises in the gastric antrum, and the combined deletion of MLH1 and PMS2 is the main cause. There was no significant difference in the Lauren classification among the patients. dMMR GC was most common in the early TNM stages, and these patients had a better prognosis. Patients with dMMR GC with vascular tumor thrombus or >6 lymph node metastases had a high recurrence rate and poor survival outcomes.

## Figures and Tables

**Table 1:** Clinical and pathological features of GC cancer patients (*n* = 162).

Clinical feature	Overall (*n* = 162)	Death (*n* = 25)	*P* value	Recurrence (*n* = 31)	*P* value
Sex			*P* = 0.678		*P* = 0.510
Male	105 (65%)	16 (64%)		19 (61%)	
Female	57 (35%)	9 (26%)		12 (39%)	
Age			*P* = 0.193		*P* = 0.875
≤55	42 (26%)	4 (16%)		8 (26%)	
>55	120 (74%)	21 (84%)		23 (74%)	
Family history^a^			*P* = 0.048^∗^		*P* = 0.310
Absent	147 (91%)	25 (100%)		29 (94%)	
Present	15 (9%)	0 (0%)		2 (6%)	
Number of tumor			*P* = 0.117		*P* = 0.253
Single	153 (94%)	22 (88%)		28 (90%)	
Multiple	9 (6%)	3 (12%)		3 (10%)	
Nerve invasion			*P* = 0.080		*P* = 0.010^∗^
Negative	117 (72%)	14 (56%)		16 (52%)	
Positive	45 (28%)	11 (44%)		15 (48%)	
Vascular tumor thrombus			*P* < 0.001^∗^		*P* < 0.001^∗^
Negative	101 (62%)	8 (32%)		11 (35%)	
Positive	61 (38%)	17 (78%)		20 (65%)	
Surgical approach			*P* = 0.887		*P* = 0.900
Laparotomy	143 (88%)	22 (88%)		28 (90%)	
Laparoscopy	19 (12%)	3 (12%)		3 (10%)	
Clinical stage^b^			*P* < 0.001^∗^		*P* < 0.001^∗^
Stage I	35 (22%)	2 (8%)		2 (6%)	
Stage II	70 (43%)	3 (12%)		4 (13%)	
Stage III	49 (30%)	16 (64%)		20 (65%)	
Stage IV	8 (5%)	4 (16%)		5 (16%)	
Number of LN^c^			*P* < 0.001^∗^		*P* < 0.001^∗^
≤6	103 (64%)	13 (52%)		15 (48%)	
>6	59 (36%)	12 (48%)		16 (52%)	
Tumor size			*P* = 0.649		*P* = 0.686
≤5 cm	103 (64%)	14 (56%)		18 (58%)	
>5 cm	59 (36%)	11 (44%)		13 (42%)	
Tumor location			*P* = 0.138		*P* = 0.352
Cardia	40 (25%)	12 (48%)		13 (42%)	
Antrum	79 (49%)	8 (32%)		12 (39%)	
Body	31 (19%)	5 (20%)		5 (16%)	
Fundus	2 (1%)	0 (0%)		0 (0%)	
Pylorus	1 (0%)	0 (0%)		0 (0%)	
Angle	9 (6%)	0 (0%)		1 (3%)	
Degree of differentiation			*P* = 0.648		*P* = 0.730
Poor	55 (34%)	10 (40%)		10 (32%)	
Mediate	48 (30%)	7 (28%)		8 (26%)	
Poor-med	55 (34%)	8 (32%)		12 (39%)	
N/A^d^	4 (2%)	0 (0%)		1 (3%)	
Lauren classification			*P* = 0.780		*P* = 0.495
Intestinal	51 (31%)	6 (24%)		7 (23%)	
Diffuse	34 (21%)	6 (24%)		8 (26%)	
Mixed	55 (34%)	9 (36%)		12 (39%)	
N/A	22 (14%)	4 (16%)		4 (13%)	
HER2			*P* = 0.025^∗^		*P* = 0.064
3+	2 (1%)	1 (4%)		1 (3%)	
0/1+/2+	156 (96%)	23 (92%)		29 (94%)	
N/A	4 (2%)	1 (4%)		1 (3%)	
dMMR^e^			*P* = 0.801		*P* = 0.512
MLH1	6 (4%)	2 (8%)		3 (10%)	
PMS2	44 (27%)	10 (40%)		11 (35%)	
MSH2	4 (2%)	0 (0%)		0 (0%)	
MSH6	9 (6%)	2 (8%)		3 (10%)	
MLH1+PMS2	93 (57%)	10 (40%)		13 (42%)	
MSH2+MSH6	5 (3%)	1 (4%)		1 (3%)	
PMS2+MSH2+MSH6	1 (1%)	0 (0%)		0 (0%)	
Treatment pattern			*P* = 0.487		*P* = 0.215
Surgery alone	84 (52%)	14 (56%)		16 (52%)	
Neoadjuvant chemotherapy	14 (9%)	4 (16%)		6 (19%)	
Postoperative adjuvant chemotherapy	61 (38%)	7 (28%)		9 (29%)	
Postoperative immunotherapy	2 (1%)	0 (0%)		0 (0%)	
Postoperative chemoradiotherapy	1 (0%)	0 (0%)		0 (0%)	

^a^History of tumorigenic first- and second-degree relatives. ^b^TNM staging classified according to the Union for International Cancer Control (UICC) TNM classification, 8th edition. For patients with multiple lesions, T staging was performed for the lesion with the highest T stage. ^c^The number of regional lymph nodes with metastases identified during surgery. ^d^N/A indicates either that testing was not done or that the results could not be evaluated. ^e^dMMR status based on the IHC staining of MMR proteins and the loss of any MMR proteins.

**Table 2:** Features and MMR protein expression levels of patients with multiple lesions (*n* = 9).

Number	Age	Family history	Personal history of tumor	MMR protein expression	Site of gastric neoplasms	Site of extragastric neoplasms	Synchronous/metachronous
01	53	Absent	No	PMS2(-)	Cardia	Esophagus	Synchronous
02	76	Absent	Breast	MLH1(-)/PMS2(-)	Cardia	Small intestine	Synchronous
03	59	Present	Rectum	MLH1(-)/PMS2(-)	Angle	Right-side colon	Synchronous and metachronous
04	62	Absent	No	MLH1(-)/PMS2(-)	Antrum	Right-side colon	Synchronous
05	55	Absent	Rectum	MSH2(-), MSH6(-)	Antrum	No	Metachronous
06	71	Absent	No	MLH1(-)/PMS2(-)	Angle/antrum	No	Synchronous
07	85	Absent	No	PMS2(-)	Antrum	No	Synchronous
08	61	Present	No	MLH1(-)/PMS2(-)	Cardia/antrum	No	Synchronous
09	62	Absent	No	PMS2(-)	Cardia/antrum	No	Synchronous

## Data Availability

The data that support the findings of this study are available on request from the corresponding author.
